# Differentiation of Induced Pluripotent Stem Cells towards Mesenchymal Stromal Cells is Hampered by Culture in 3D Hydrogels

**DOI:** 10.1038/s41598-019-51911-5

**Published:** 2019-10-30

**Authors:** Roman Goetzke, Hans Keijdener, Julia Franzen, Alina Ostrowska, Selina Nüchtern, Petra Mela, Wolfgang Wagner

**Affiliations:** 1 Helmholtz Institute for Biomedical Engineering, Stem Cell Biology and Cellular Engineering, RWTH Aachen University Medical School, Aachen, Germany; 2 Department of Biohybrid & Medical Textiles (BioTex), AME-Institute of Applied Medical Engineering, Helmholtz Institute, RWTH Aachen University, Aachen, Germany; 3 Medical Materials and Implants, Department of Mechanical Engineering and Munich School of BioEngineering, Technical University of Munich, Garching, Germany; 4 Institute for Biomedical Engineering – Cell Biology, RWTH Aachen University Medical School, Aachen, Germany

**Keywords:** Stem-cell differentiation, Biomaterials - cells, Mesenchymal stem cells, Induced pluripotent stem cells

## Abstract

Directed differentiation of induced pluripotent stem cells (iPSCs) towards specific lineages remains a major challenge in regenerative medicine, while there is a growing perception that this process can be influenced by the three-dimensional environment. In this study, we investigated whether iPSCs can differentiate towards mesenchymal stromal cells (MSCs) when embedded into fibrin hydrogels to enable a one-step differentiation procedure within a scaffold. Differentiation of iPSCs on tissue culture plastic or on top of fibrin hydrogels resulted in a typical MSC-like phenotype. In contrast, iPSCs embedded into fibrin gel gave rise to much smaller cells with heterogeneous growth patterns, absence of fibronectin, faint expression of CD73 and CD105, and reduced differentiation potential towards osteogenic and adipogenic lineage. Transcriptomic analysis demonstrated that characteristic genes for MSCs and extracellular matrix were upregulated on flat substrates, whereas genes of neural development were upregulated in 3D culture. Furthermore, the 3D culture had major effects on DNA methylation profiles, particularly within genes for neuronal and cardiovascular development, while there was no evidence for epigenetic maturation towards MSCs. Taken together, iPSCs could be differentiated towards MSCs on tissue culture plastic or on a flat fibrin hydrogel. In contrast, the differentiation process was heterogeneous and not directed towards MSCs when iPSCs were embedded into the hydrogel.

## Introduction

Induced pluripotent stem cells (iPSCs) hold enormous potential for regenerative medicine^1^. In principle, they can give rise to all cell types of our body, but it remains a challenge to direct cell fate decisions under *in vitro* conditions towards specific cell types. Of particular relevance is the directed differentiation of iPSCs towards mesenchymal stromal cells (MSCs), which are used in a multitude of clinical trials and for tissue engineering^2^. Such iPSC-derived MSCs might overcome several limitations observed with natural MSCs: i) primary MSCs are rare within tissues and not easily accessible *in vivo*^3^; ii) they are highly heterogeneous and vary extensively between different tissues and donors^4^; and iii) culture expansion of MSCs is limited by replicative senescence^5^. In contrast, iPSCs reflect a ground state of pluripotency without any signs of cellular aging. This unrestricted starting material might thus give rise to more homogeneous and standardized MSC-like cell preparations.

So far, differentiation of iPSCs towards MSCs (iMSCs) was performed on tissue culture plastic (TCP). To induce differentiation towards this lineage, the culture medium is supplemented with either human platelet lysate (hPL)^6^, or fetal calf serum (FCS)^7^. hPL contains a large spectrum of cytokines, growth factors, and mitogenic compounds that promote *in vitro* MSC expansion^8,9^. Accordingly, iMSCs generated with hPL enriched medium fulfill the minimal criteria for the definition of MSCs^10^. However, there are large differences between iMSCs and primary MSCs on epigenetic level, indicating that the differentiation regimen needs to be further optimized^6^. The relevance of matrix elasticity for directed differentiation has been described before^11^. In our previous work, we have therefore compared iMSCs that were either generated on TCP or on a very soft hydrogel consisting of human platelet lysate^12^. To our surprise, generation of iMSCs was hardly influenced by the underlying substrate. There were no clear differences in growth, morphology, *in vitro* differentiation, gene expression profiles, and DNA methylation (DNAm) patterns if iMSCs were generated either on TCP or on hydrogel. Thus, matrix elasticity alone might not be sufficient to promote lineage-specific differentiation of iPSCs into genuine MSCs^12^.

Another important parameter might be the three-dimensional (3D) microenvironment that can mimic extracellular matrix properties of native tissue^13^. Hydrogels composed of natural components, such as collagen^14^, or fibrin^15^, provide integrin binding sites (e.g. RGD-motifs) to support cell adhesion and migration^16^. Moreover, 3D scaffolds have different physical and biochemical cues, which affect differentiation of MSCs^17^. Thus, hydrogels are bioactive materials that might also impact on regulation of differentiation processes of iPSCs^18^, but the relevance of 3D scaffolds for generation of iMSCs has not yet been addressed.

Fibrin forms during blood clotting by reaction of the two coagulation factors fibrinogen and thrombin^19^. This natural polymer cross-links very rapidly, allowing encapsulation of cells *in vitro*^20^, and it can be lysed efficiently with various enzymes^21–23^. Furthermore, fibrin is exploited in tissue engineering^24,25^, cell delivery^26,27^, drug delivery applications^28,29^, and even in cancer research^30–32^. Fibrin gel has been widely used as a scaffold for MSCs, as it improves their therapeutic effect by sustaining growth and function^33,34^, and it supports *in vivo* transplantation of MSCs^35,36^. Furthermore, fibrin hydrogels have been seeded with iPSCs^37,38^, but it is yet unclear how 3D scaffolds impact on differentiation towards MSCs.

In this study, we compared iPSC differentiation towards MSCs on conventional tissue culture plastic, on flat fibrin gel, or inside a 3D fibrin gel. Differentiation was assessed morphologically using two-photon microscopy and by flow cytometric analysis of MSC surface markers. Furthermore, we analyzed global gene expression and DNA methylation profiles to assess molecular changes that occurred during differentiation. We demonstrate that iPSCs proliferated, migrated, and differentiated within fibrin hydrogel for several weeks without passaging. However, in contrast to differentiation on flat substrates, the 3D culture conditions impaired differentiation towards an MSC-like phenotype.

## Results

### Comparison of iMSC generation in 2D and 3D culture with fibrin gel

Rheological measurements demonstrated that the fibrin hydrogels had an elastic modulus of ~700 Pa (Suppl. Fig. S1). The typical strain-dependent stiffening of fibrin gel was observed^12,39^. In contrast, the maximum viscous modulus was only about 150 Pa. Thus, our fibrin hydrogel revealed viscoelastic properties with a relatively high elastic modulus.

**Figure 1:**
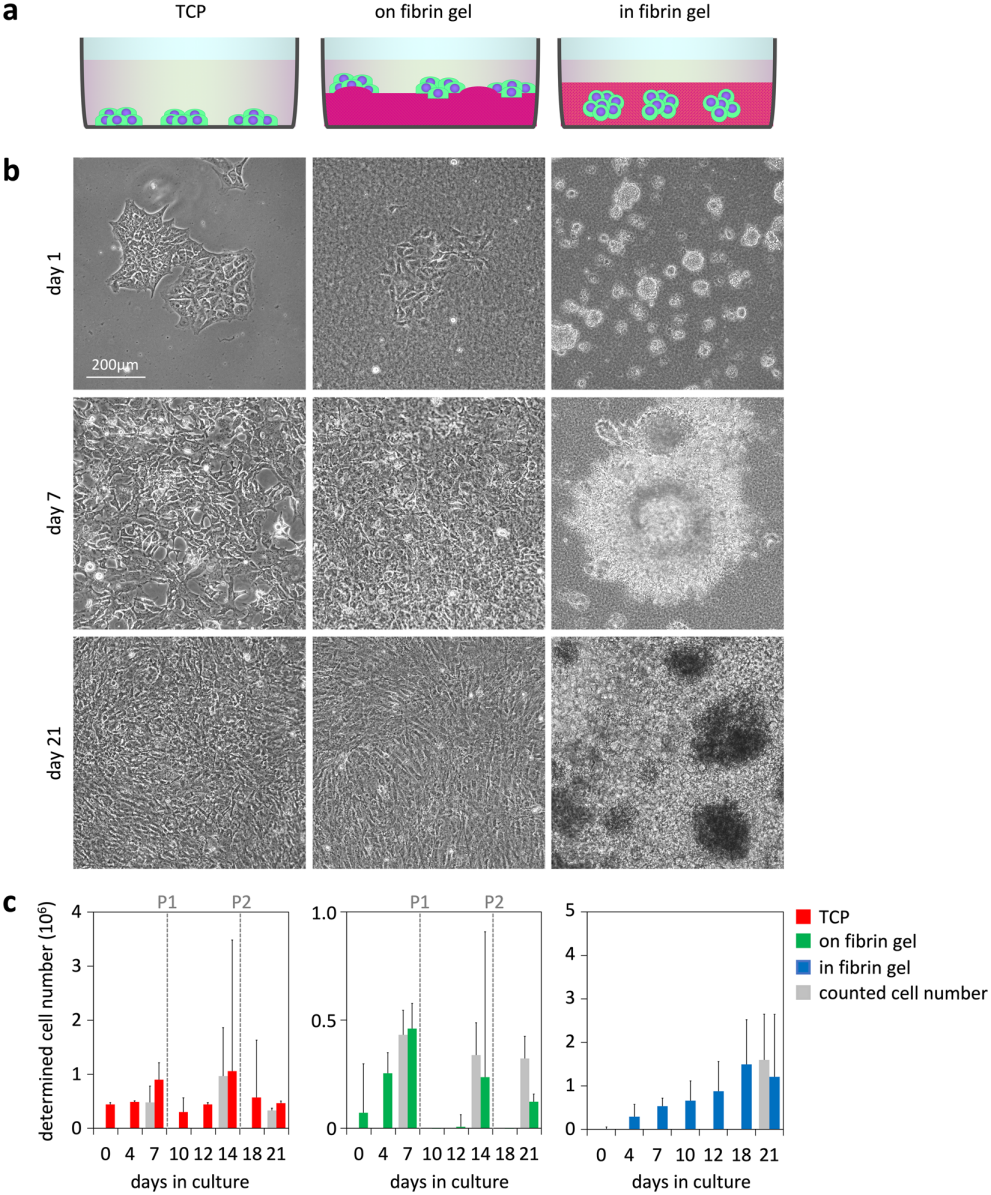
Differentiation of iPSCs on flat substrates and within hydrogel. (**a**) Schematic presentation of differentiation of iPSCs on tissue culture plastic (TCP), on fibrin gel, or within fibrin gel. (**b**) Phase contrast images in the course of differentiation. After 21 days, the cells on flat substrates showed MSC-like morphology, whereas the colonies within the hydrogel enlarged with heterogeneous growth patterns. (**c**) Proliferation of three differentiated iPSC lines was estimated with a non-methylated reference DNA^42^. The results were validated for selected time points by direct counting of cells in a counting chamber (indicated in grey). Standard deviation was calculated from the three independent iPSC lines.

Subsequently, we analyzed if fibrin hydrogel supports differentiation of iPSCs towards MSCs. To this end, we seeded iPSCs in parallel in three different culture conditions (Fig. 1a): i) as a reference, we used tissue culture plastic (TCP) for differentiation of iPSCs towards MSCs; ii) fibrin gel was used as a flat substrate; and iii) iPSCs were embedded into fibrin gel. Differentiation towards MSCs was induced by switching to culture medium with 10% hPL (hPL-medium), as described before^6^. Addition of ROCK inhibitor was required to inhibit apoptosis of iPSCs in 3D culture^40^. Since ROCK inhibitor can prime iPSC differentiation towards mesendodermal lineage^41^, we applied the same concentration to all of our culture conditions. On TCP, the cells revealed continuous morphological changes and acquired a typical MSC-like growth pattern after 21 days of differentiation. The same morphological changes were also observed if cells were grown on top of the fibrin gel, while we did not observe cell migration into the hydrogel. In contrast, iPSC colonies that were embedded into fibrin gel inflated into sphere-like structures within the first week of differentiation (Fig. 1b). These spheres gave rise to highly proliferative, spindle-shaped cells that grew out from the surface of the spheres between 7–14 days of differentiation. These cells migrated through the gel, albeit there were regions with higher and lower cell density after 21 days of differentiation. We have not observed cells migrating towards the surface of the gel while the cells were embedded in fibrin gel.

Quantification of cell proliferation in 3D hydrogels is not trivial due to cell loss when harvesting. We therefore adopted an innovative approach to estimate cell numbers based on a reference DNA (Suppl. Fig. S2a). This reference DNA comprised a non-methylated sequence, which is methylated in the cellular DNA. So far, the method has only been described for quantification of leucocytes^42^. In fact, genomic DNA of iPSCs and MSCs was also consistently highly methylated at the relevant loci of the genes *LSM14B* and *ZC3H3*. When we mixed different numbers of iPSCs with the same amount of a reference plasmid, the pyrosequencing results revealed a very high correlation of DNAm and cell numbers (R = 0.98; Suppl. Fig. S2b). Furthermore, the estimated cell numbers based on DNAm measurements were in a similar range as observed with a counting chamber (Fig. 1c). Some of the variation can be attributed to differences in cell attachment and growth between different iPSC lines used in this study. Notably, in comparison to the conventional cell counting, our approach was feasible with much smaller samples (one 24-well) and it was not affected by cell ruptures or cell death during harvesting from the gels. Taken together, the results validated continuous proliferation of cells embedded into fibrin hydrogel.

### Differentiation within fibrin hydrogel results in heterogeneous growth patterns

**Figure 2:**
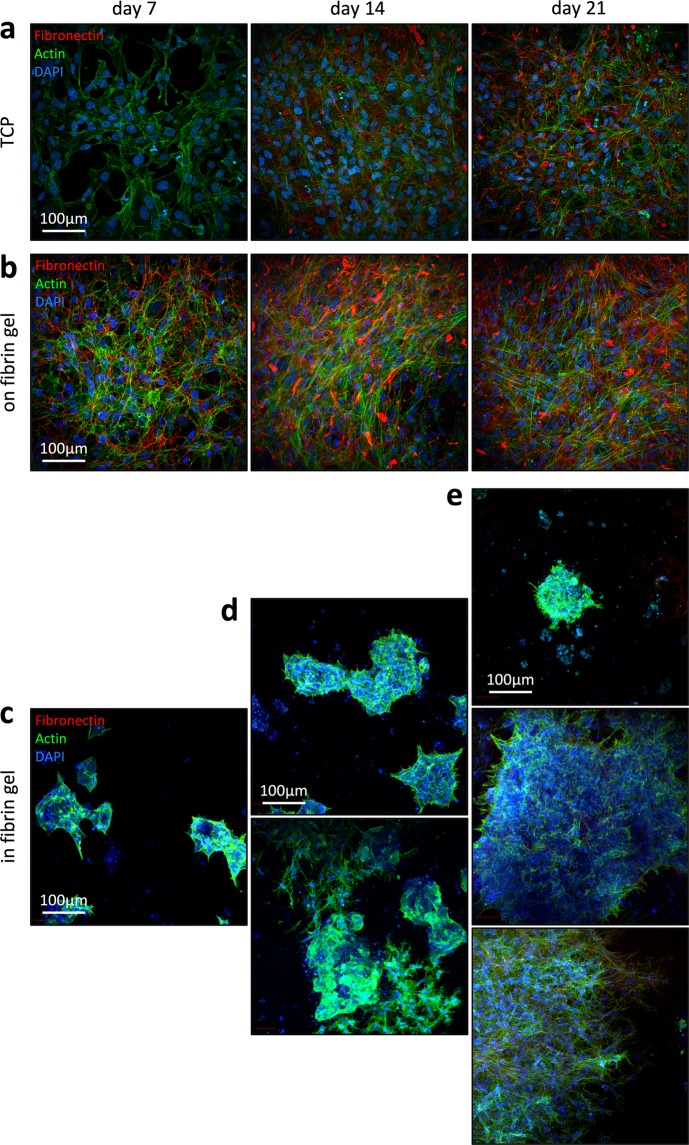
Growth patterns of iPSC-derived cells in different culture conditions. The fibronectin deposition (red) and actin organization (green) was analyzed with two-photon microscopy at different time points (7, 14, or 21 days) on TCP (**a**), on fibrin gel (**b**), and in fibrin gel (**c**,**d**). Nuclei were counterstained with DAPI (blue).

Subsequently, we analyzed the growth pattern, fibronectin secretion, and cytoskeletal organization with two-photon microscopy. On TCP, the cells enlarged and elongated during generation of iMSCs. Furthermore, the cells secreted fibronectin, which formed a dense extracellular network without being organized in line with the thin actin stress fibres in the cells (Fig. 2a). Differentiation of iPSCs on top of fibrin hydrogel resulted in even more pronounced deposition of fibronectin and higher migratory activity (Fig. 2b). In contrast, the iPSCs that were embedded into hydrogel remained as circumscribed and relatively small colonies within the first week (Fig. 2c). After one week of differentiation in 3D, cavities filled with apoptotic cells could be observed inside the colonies (Suppl. Fig. S3), as previously described for embryoid bodies^43^. During the second week of 3D cultivation, a clear heterogeneity emerged between these clusters: some colonies remained compact with an apoptotic cavity, other colonies displayed a star-like growth pattern and highly migratory, elongated cells (Fig. 2d). This heterogeneity became even more pronounced at day 21 of differentiation, when some colonies remained small and compact, others grew into large densely packed conglomerates, or into outspread large colonies, where the migratory cells seemed to have a similar organization of actin fibres, as observed for iMSCs (Fig. 2e). Notably, none of these colonies within hydrogel revealed a clear fibronectin expression. Only the highly migratory fibroblastoid colonies revealed faint fibronectin filaments after three weeks. Taken together, in contrast to differentiation on flat substrates, the differentiation of iPSCs within fibrin hydrogel resulted in highly heterogeneous growth patterns.

### Cell characterization by immunophenotype and *in vitro* differentiation potential

**Figure 3:**
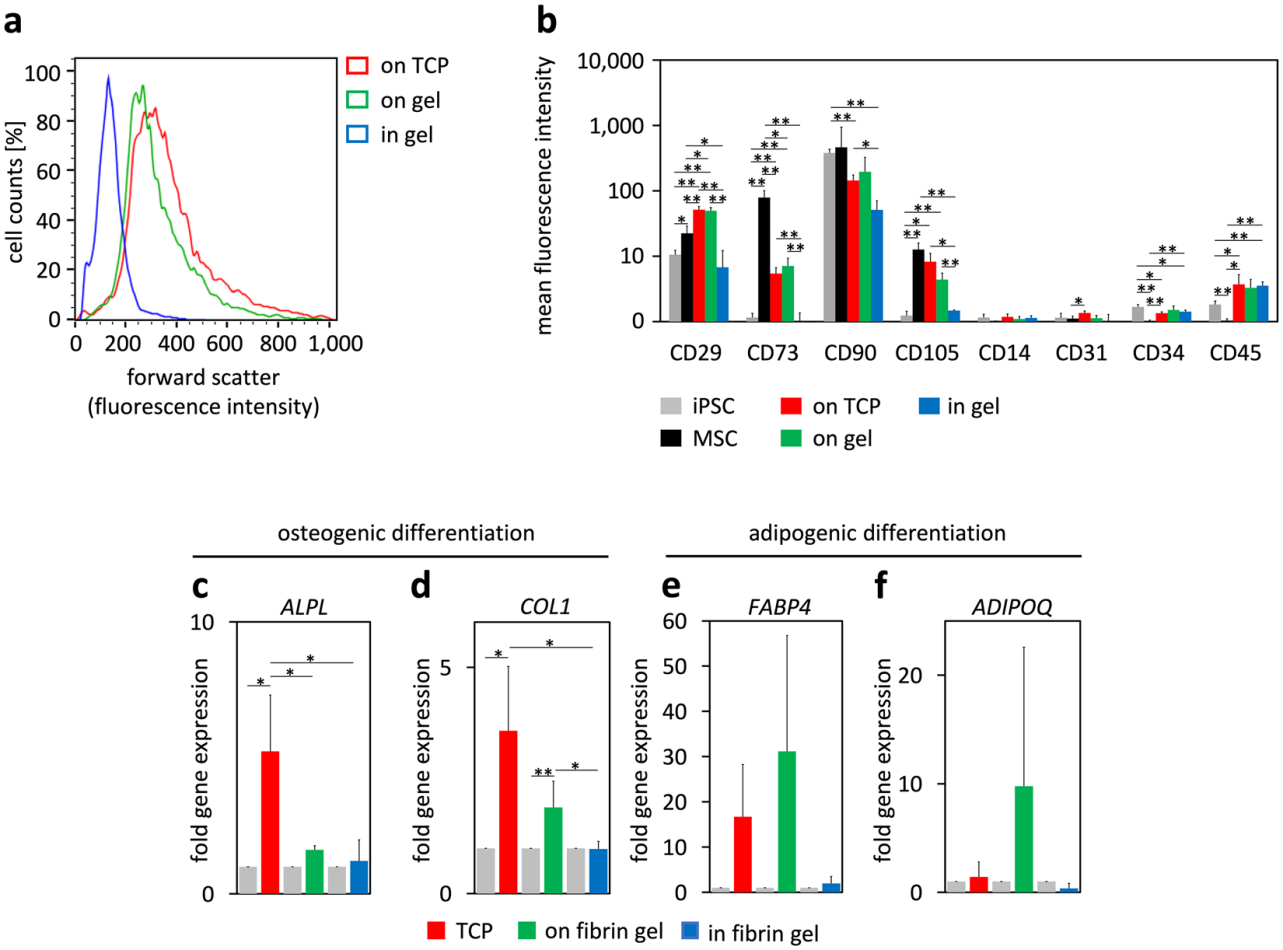
Immunophenotype and differentiation potential of iPSC-derived cells. (**a**) Forward scatter in flow cytometric analysis indicated that after 21 days of differentiation the cells were much smaller if they were differentiated in hydrogel as compared to culture on flat substrates. (**b**) Differentiation of iPSCs on TCP or on fibrin gel resulted in a similar immunophenotype as MSCs, whereas particularly CD73 and CD105 were not expressed upon differentiation within fibrin gel (mean fluorescence intensity normalized to autofluorescence ± SD, n = 3, p-value adjusted for multiple testing: *p < 0.05, **p < 0.01). (**c**–**f**) After 14 days, up-regulation of osteogenic (*ALPL*, *COL1*) and adipogenic (*FABP4*, *ADIPOQ*) marker genes was analyzed by qRT-PCR (n = 3; *p < 0.05, **p < 0.01).

Flow cytometric analysis was compared after iPSC differentiation for three weeks on TCP, on fibrin gel, or within the fibrin hydrogel. The significantly smaller forward scatter supported the notion that cells, which were generated in fibrin gel, remained much smaller (Fig. 3a). When iPSCs were differentiated on TCP or on fibrin gel, they displayed a very similar immunophenotype as primary MSCs (CD29^+^; CD73^+^; CD90^+^; CD105^+^, CD14^−^; CD31^−^; CD34^−^, and CD45^−^; Fig. 3b and Suppl. Fig. S4). However, when iPSCs were cultivated embedded into fibrin hydrogel, upregulation of CD73 and CD105 was almost undetectable, similar to iPSCs. Thus, generation of iMSCs on flat substrates resulted in an immunophenotype, which closely resembled primary MSCs, whereas this was not observed if the cells were differentiated in the hydrogel.

We subsequently induced osteogenic and adipogenic differentiation of iPSC-derived cells while the cells were still cultured on the flat substrates or within the fibrin gel (chondrogenic differentiation was not considered, because it is performed in pellet culture). As expected by the different matrix elasticity, osteogenic differentiation was enhanced on TCP, while adipogenic differentiation appeared more pronounced on the softer fibrin gel^11,44^. In contrast, when osteogenic differentiation was induced in iPSC-derived cells within fibrin gel we did not observe up-regulation of osteogenic marker genes (*ALPL*, *COL1*) (Fig. 3c,d), while analysis of alkaline phosphatase staining was hampered by the 3D hydrogel (Suppl. Fig. S5). In analogy, adipogenic differentiation within fibrin gel did not result in up-regulation of adipogenic marker genes (*FABP4*, *ADIPOQ*) (Fig. 3e,f), and the cells did not acquire the typical intracellular fat droplets (Suppl. Fig. S5). These results support the notion that differentiation of iPSCs was not directed towards MSCs during culture in a 3D fibrin matrix.

To facilitate better comparison of the *in vitro* differentiation potential, including chondrogenic differentiation, we have alternatively harvested the cells after 21 days from the three different culture conditions and induced three-lineage differentiation in parallel on cell culture plastic. Staining with Alizarin Red, BODIPY, and Alcian Blue indicated that all cell preparations could now be differentiated towards osteocytes, adipocytes, and chondrocytes, respectively (Suppl. Fig. S6a). This three-lineage differentiation potential of iMSCs was further validated on gene expression levels by up-regulation of lineage-specific markers (Suppl. Fig. S6b–g). It was somewhat unexpected, that iPSC-derived cells, which were initially differentiated within fibrin gel, revealed the same multilineage differentiation potential as iMSCs that were generated on TCP or on fibrin gel. Furthermore, all iPSC-derived cells adopted to the same typical fibroblastoid morphology and growth pattern upon reseeding on TCP. Thus, all iPSC-derived cells, even those that were isolated from 3D hydrogels, apparently assimilated towards the same cell type, when they were reseeded on the TCP for the additional three-lineage differentiation.

### Differentiation within hydrogel hampers upregulation of extracellular matrix- and MSC-associated genes

**Figure 4:**
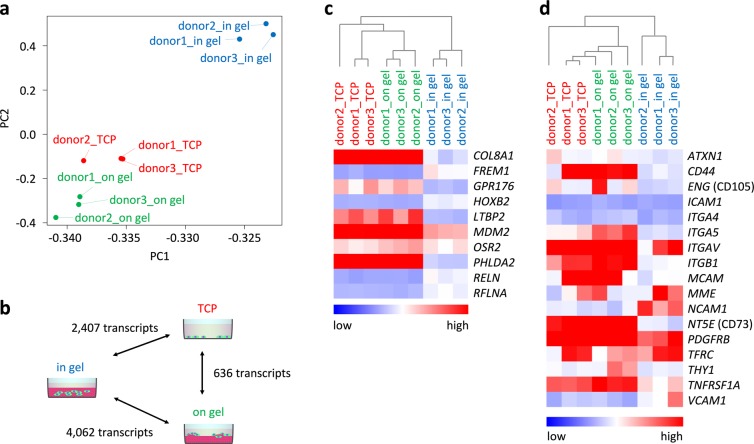
Gene expression profiles are affected by different substrates. Upon differentiation of iPSCs (donors 1–3) for three weeks on TCP, on fibrin gel, and within fibrin gel, the transcriptome was analyzed by RNA-seq. (**a**) Principal component analysis (PCA) demonstrated that particularly differentiation in hydrogel resulted in different gene expression profiles. (**b**) Pairwise comparison of differential gene expression. The number of significant transcripts is indicated (adjusted p-value < 0.05 and at least two-fold differential expression). (**c**) Heatmap depicts relative gene expression levels of the ten most significantly differentially expressed genes between 2D *versus* 3D (adjusted p-value < 5 × 10^−28^). (**d**) Heatmap depicts relative gene expression levels of genes that are typically expressed in MSCs.

We then analyzed how differentiation on flat substrates or in hydrogel is reflected in the transcriptome. After 21 days of differentiation, cells were harvested from TCP, from the surface of fibrin hydrogel, or from within the hydrogel and analyzed by RNA-seq (n = 3). Principal component analysis (PCA) clearly separated cells according to the three substrates for differentiation (Fig. 4a). Pairwise comparisons demonstrated many significant differences in gene expression, particularly between cells that were generated on flat substrates *versus* within the hydrogel (adjusted p-value < 0.05; in addition, a cut-off of two-fold differential expression was applied; Fig. 4b). Gene Ontology demonstrated that in the 3D culture conditions transcripts involved in neurogenesis were rather upregulated, whereas genes associated with wound healing and angiogenesis were down-regulated (Suppl. Fig. S7). Amongst the ten most significant gene expression changes between 2D *versus* 3D culture were collagen 8A1 (*COL8A1*), FRAS1 related extracellular matrix 1 (*FREM1*), latent transforming growth factor beta binding protein 2 (*LTBP2*), reelin (*RELN*), odd-skipped related transciption factor 2 (*OSR2*), and the regulator of the actin cytoskeleton refilin A (*RFLNA*; Fig. 4c). In fact, genes associated with the actin cytoskeleton and ECM were generally less expressed if iPSCs were differentiated within the fibrin hydrogels, and this was also observed for fibronectin as described above (Suppl. Fig. S8). Notably, MSC-associated genes, including endoglin (*ENG* or CD105) and ecto-5-nucleotidase (*NT5E* or CD73), were much higher expressed if iMSCs were differentiated on the flat substrates (Fig. 4d). These results support the notion that differentiation of iPSCs within the fibrin hydrogel hampered differentiation towards MSCs.

### Epigenetic profiles upon differentiation in 2D *versus* 3D culture conditions

**Figure 5:**
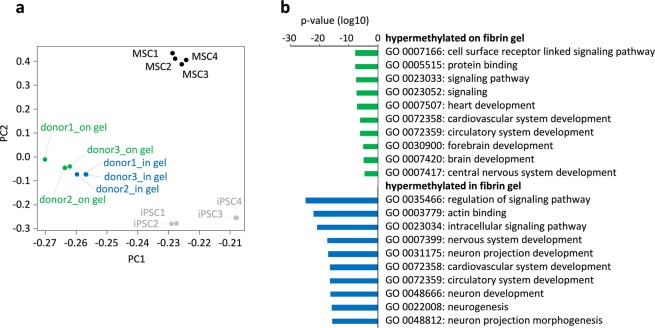
Differentiation of iPSCs either on or within hydrogel results in different DNA methylation patterns. After three weeks of differentiation of iPSCs (donors 1–3) either on fibrin gel or within fibrin gel, the DNA methylation profiles of the resulting cells were analyzed with Illumina Infinium MethylationEPIC BeadChips. (**a**) Principal component analysis showed clear separation of the cell types. For comparison, we have also included DNA methylation profiles of MSCs (GSE113527) and iPSCs (GSE95531). (**b**) 13,352 CpGs revealed significant differences in DNAm between iPSC-derived cells in 2D and 3D. The corresponding genes were further classified by Gene Ontology analysis.

The phenotypic differences of cells in different culture conditions might either be transient, or they might be determined by epigenetic modifications. To address this question, we compared global DNA methylation (DNAm) profiles of the iPSC-derived cells that were differentiated either on the fibrin gel or within the fibrin hydrogel. For comparison, we utilized available data sets of MSCs and iPSCs (Table S1). Epi-Pluri-Score analysis validated that all iPSC-derived cells were not classified as pluripotent cells any more upon differentiation^45^ (Suppl. Fig. S9). Principal component analysis clearly separated all cell preparations, indicating that there are marked epigenetic differences (Fig. 5a). 13,352 CpGs revealed significant differences upon differentiation in 2D *versus* 3D conditions (adjusted p-value < 0.05; in addition, a cut-off of 20% differential methylation was applied). These differentially methylated CpGs were particularly enriched in genes of the Gene Ontology categories for cardiovascular system and neurogenesis, respectively (Fig. 5b). Thus, differentiation of iPSCs under either 2D or 3D culture conditions had marked and reproducible impact on the epigenetic markup.

## Discussion

One of the commonly used criteria for definition of mesenchymal stromal cells is their plastic adherent growth^10^, whereas the cells are embedded into a 3D connective tissue in their natural environment. MSCs are largely defined by a uniform and directed growth pattern, which is observed on flat polystyrene surfaces. However, little is known about the growth behavior of their *in vivo* counterpart. Thus, the concept of MSCs is relatively ill defined^46^ and more physiologic culture systems are needed to study the development of the various mesodermal derivatives, which are summarized under this term^47^.

Several studies have demonstrated that primary MSCs can be cultured in hydrogels and that such culture conditions might even support their *in vitro* differentiation towards osteogenic^48^, adipogenic^49,50^, and chondrogenic lineage^51,52^, or into smooth muscle cells^53^. In contrast to these studies, we analyzed how culture within a hydrogel impacts on the differentiation of iPSCs towards MSCs. We did not aim for an understanding of how fibrin hydrogel affects iMSCs, but how the process of differentiation towards this cell type is supported within a hydrogel. In fact, the DNA methylation patterns of iPSC-derived cells cultured under different conditions revealed highly significant and reproducible differences. These results demonstrate that differentiation on flat fibrin gel *versus* embedded into fibrin gel results in a very different cell fate that is determined by epigenetic means.

Defined three-dimensional microenvironments have been used for culture expansion of iPSCs before^54^ and they might even boost induction of pluripotency^55^. Furthermore, differentiation of iPSCs in 3D cell culture regimen has emerged as a model system for investigating human embryonic development and disease progression *in vitro*. Self-organizing structures derived from the pluripotent cells are commonly termed “organoids” and recapitulate many aspects of the structural organization of *in vivo* organ counterparts^56^. Embryoid bodies are marked by apoptosis-mediated cavitation during epithelial morphogenesis^43^ and a similar process might result in the lumen of our iPSC-derived colonies within fibrin hydrogels. The heterogeneity of iPSC-colonies within the fibrin gel might arise from self-organizing processes with randomly taken cell fate decisions. Either way, the characteristic surface markers for MSCs and genes that are usually expressed in MSCs were hardly expressed in any of the cells that were differentiated in 3D.

In this study, we have chosen a fibrin hydrogel for 3D culture expansion, because this natural polymer is widely used in tissue engineering. Culture within 3D fibrin gel did not require weekly passaging, due to the continuous growth and migration of cells within the hydrogel. It is known that passaging using trypsin causes severe cell stress and alters the physiology of mammalian cells^57^. Therefore, we anticipated that a one-step procedure for iPSC differentiation towards MSCs directly within the fibrin scaffold would reduce cell stress and, due to the embedding in extracellular matrix, ease their application in tissue engineering. It is conceivable, that the need of passaging on flat substrates contributes to the differences as compared to cells embedded into fibrin gel – but it appears unlikely that passaging is the relevant stimulus for cell-type specific differentiation towards MSCs. Furthermore, we were able to directly compare the cellular progeny under 2D and 3D conditions with the identical substrate and elasticity. In fact, the iPSC-derived cells proliferated and migrated within the fibrin hydrogel. Notably, these cells within hydrogel hardly secreted extracellular matrix, such as fibronectin, whereas primary MSCs secrete such components if embedded into hydrogels^12^. In the future, other hydrogels might be tested, but due to the labor-intensive experiments with different iPSC-clones and sophisticated molecular characterization, we have focused in this study on the matrix that appeared to be best suited.

Matrix elasticity of fibrin scaffolds is similar to other soft substrates that are widely exploited to promote culture of neuronal cells and to induce neuronal lineage commitment in iPSCs^38,58^. In fact, when iPSCs were differentiated within this hydrogel, particularly genes involved in neurogenesis were upregulated. Furthermore, the overall enhanced DNA methylation in transcriptional regions of genes involved in neuronal development indicates that such genes are epigenetically activated in 3D conditions. However, due to the complexity of stimuli within a 3D elastic matrix it is hard to grasp, which factors caused the differences in cell fate decisions^12^. For instance, it has been demonstrated that the high degree of cross-linking inside a polymer network decreases diffusion of water, ions, and small molecules which might interfere with cell growth^59^. Furthermore, oxygen pressure has been shown to be an influencing factor in the differentiation process of stem cells^60,61^. However, as the fibrin gels that we generated were very thin (1–2 mm in depth), it is unlikely that the cells inside fibrin gel experienced severe hypoxic conditions. Furthermore, we have recently shown that matrix elasticity alone does not impact the differentiation process of iPSCs towards MSCs^12^. In this study, we observed moderate differences in gene expression profiles if iPSCs were differentiated on rigid TCP or on fibrin hydrogel, and this might also be due to other differences between the substrates or regimen for passaging.

Taken together, differentiation on flat substrates resulted in iMSCs that closely resembled primary MSCs in function, phenotype, and gene expression profile. In contrast, a 3D differentiation regimen within fibrin hydrogel did not trigger differentiation towards this phenotype. Therefore, the 3D culture condition within the hydrogel clearly influenced cell differentiation of iPSCs, resulting in cells that are markedly different from those derived on flat substrates.

## Methods

### Cell culture

Mesenchymal stromal cells were isolated from femoral bone marrow of patients undergoing orthopedic surgery as described before^62^. All samples were taken after informed and written consent using guidelines approved by the Ethic Committee for the Use of Human Subjects at the University of Aachen (permit number: EK128/09). All methods were performed in accordance with the relevant guidelines and regulations. For all experiments, we used three independent iPSC lines that were generated as described before^63^. In brief, MSCs were reprogrammed with episomal plasmids into iPSCs, subcloned, and cultured on TCP coated with vitronectin (0.5 µg/cm^2^) in StemMACS iPS-Brew XF (all Miltenyi Biotec GmbH, Bergisch Gladbach, Germany). Pluripotency was validated by *in vitro* differentiation and Epi-Pluri-Score^45^ (Cygenia GmbH, Aachen, Germany).

To differentiate iPSCs towards MSCs, the culture medium was exchanged for Dulbecco ´s modified Eagle ´s medium-low glucose (DMEM-LG, Gibco/Thermo Fisher Scientific, Darmstadt, Germany) supplemented with 10% human platelet lysate (hPL-medium) for optimal cell growth. hPL was prepared from expired thrombocyte units, as described in detail before^9^. Furthermore, the medium was supplemented with 5 U/ml heparin (Rotexmedica, Trittau, Germany), 10 µM of the Rho-associated protein kinase (ROCK) inhibitor Y-27632 (Abcam, Cambridge, UK), and 250 U/ml aprotinin (Nordic Pharma, Reading, UK). For culture on TCP, the cells were always passaged after 7 days using trypsin-EDTA 0.25%^6^ (Gibco/Thermo Fisher Scientific); for culture on top of the fibrin hydrogel, the cells were passaged every 7 days by lysis of gels with 50 fibrin degradation units (FU)/ml nattokinase (dissolved in PBS; NSK-SD; Japan Bio Science Laboratory Co., Osaka, Japan) for 30 min at 37 °C and the cells were subsequently separated with trypsin-EDTA 0.25%; if cells were embedded into fibrin hydrogels, passaging was not required within the first three weeks due to migratory activity without contact inhibition. iMSCs were harvested after 21 days of differentiation under the different culture conditions for systematic comparison.

### Fibrin hydrogel

Fibrin gel consisted of fibrinogen (5 mg/ml), CaCl_2_ (3,75 mM), and thrombin (20 U/ml); all fibrin gel components were dissolved in tris-buffered saline (TBS)-buffer composed of Tris-Cl (50 mM), NaCl (150 mM), and deionized water (pH 7.5; all Sigma Aldrich, Hamburg, Germany). For 2D culture on fibrin gel, 25,000 iPSC colonies were seeded directly on top of fibrin gel (1 ml) inside a 6-well in MSC culture medium (supplemented with hPL, heparin, ROCK inhibitor, and aprotinin, as described above). For 3D culture, the cells were embedded into fibrin gel. In brief, we prepared a bottom fibrin gel (1 ml) inside a 6-well (10 cm^2^). After polymerization, 25,000 iPSC colonies were resuspended in fibrinogen solution (500 µl of 5 mg/ml fibrinogen in TBS-buffer), which polymerized on top of the freshly prepared fibrin gel, and overlaid by an additional 1 ml fibrin gel. Each fibrin gel layer polymerized for 10 min at 37 °C. Due to embedding of cells between the two layers of fibrin gel it was negligible that stiffness of the gel might be higher close to the bottom of the cell culture well^64^. The fibrin gel was overlaid with StemMACS iPS-Brew XF (1.5 ml) with heparin (5 U/ml), ROCK-inhibitor (10 µM), and aprotinin (250 U/ml) for 24 h, before changing to MSC culture medium.

### Rheological measurements

Rheology was performed with a Kinexus ultra+ rheometer (Malvern Instruments, Worcestershire, UK) equipped with a cone geometry (model CP4/40 SR2938; 40 mm Ø). The bottom plate of the device was cooled to 4 °C before 150 µl of thrombin and fibrinogen solution (as described above) were pipetted on top of the plate. The gel polymerized for 30 min at 37 °C. Elastic and viscous moduli of fibrin gel were analyzed at 37 °C with controlled strain sweeps at 1 Hz from 0.1–100% strain^12^.

### Proliferation analysis

To estimate proliferation within the hydrogel, we adopted a recently described method based on DNA methylation^42^. In brief, all cells (with or without hydrogel) were harvested and mixed with a reference plasmid comprising the non-methylated sequence of either *LSM14B* (0.022 µg) or *ZC3H3* (0.011 µg). The genomic DNA was then isolated with the NucleoSpin Tissue kit (Macherey-Nagel, Düren, Germany). DNAm levels of the relevant single CpG sites in *LSM14B* (cg06096175) and *ZC3H3* (cg25834632) were analyzed by pyrosequencing^42^. These relevant genomic regions are methylated in iPSCs and MSCs, whereas they are non-methylated in the reference plasmid. The copy number of the reference plasmid (*C*_*R*_) can therefore be determined with the following formula:

$${C}_{R}=1.5\cdot \frac{{m}_{R}\cdot {N}_{A}}{MW}$$

where *m*_*R*_ is the added reference amount, *N*_*A*_ is Avogadro’s constant; and *MW* is the molar weight of the reference DNA (for *LSM14B*: 2.85 × 10^−6^ gmol^−1^; for *ZC3H3*: 2.88 × 10^−6^ gmol^−1^). The cell numbers can then be estimated with the following formula:

$$\frac{{\rm{cells}}}{\mu {\rm{l}}}=\frac{{C}_{R}\cdot ({\rm{DNAm}}-{\rm{a}})}{2\cdot ({\rm{b}}-{\rm{DNAm}})}$$

where DNAm is the measured DNA methylation of the sample; and a and b are absolute DNAm levels of either pure reference DNA or genomic DNA, respectively.

### Fluorescence staining and two photon scanning microscopy

Samples were fixed with 4% paraformaldehyde for 30 min; blocked with 5% normal goat serum (NGS); and permeabilized with 0.1% triton-x (Bio-Rad, München, Germany) for 1 h. Cells were stained with anti-fibronectin antibody (clone A-11; Santa Cruz Biotechnology, Dallas, USA); actin was stained using Alexa-488 conjugated phalloidin (BioMol, Hamburg, Germany); and nuclei were counterstained with DAPI (10 ng/ml) for 10 min. Samples were analyzed with a FV1000MPE two-photon microscope to investigate 3D growth (Olympus Corp., Tokyo, Japan). To analyze viability of iPSC colonies in fibrin gel, we stained living cells with Calcein-AM and dead cells with propidium iodide (PI) (both Sigma Aldrich).

### Flow cytometric analysis

Immunophenotypic analysis of iPSCs, MSCs, and iMSCs was performed as described before^65^. In brief, each cell preparation was stained in parallel with CD14-APC (clone M5E2), CD29-PE (clone MAR4), CD31-PE (clone WM59), CD34-APC (clone 8G12), CD45-APC (clone HI30), CD73-PE (clone AD2), CD90-APC (clone 5E10; all BD Bioscience, Heidelberg, Germany), and CD105-FITC (clone MAR-226; ImmunoTools, Friesoythe, Germany). Flow cytometry was performed with a FACS Canto II cytometer (BD Bioscience) and data were further processed with FlowJo (FlowJo LLC, Ashland, Oregon).

### *In vitro* differentiation of iMSCs

To directly compare the three-lineage differentiation potential, the iMSCs were either harvested from the different culture conditions at day 21 and reseeded in parallel on TCP, or differentiated directly in their respective culture condition. Induction of osteogenic, adipogenic, and chondrogenic differentiation was performed as previously described^66^. In brief, after 2 weeks, osteogenic differentiation of iMSCs on TCP was analyzed by staining of calcium precipitates with Alizarin Red S^67^. Alternatively, osteogenic differentiation was analyzed by staining of alkaline phosphatase with NBT (nitro-blue tetrazolium chloride) and BCIP (5-bromo-4-chloro-30-indolyphosphate p-toluidine salt; Sigma Aldrich); NBT/BCIP does not stain fibrin gel and therefore facilitated analysis of osteogenic differentiation on and in fibrin gel. In parallel, fat droplets in adipogenic differentiation were stained with BODIPY (4,4-difluoro-1,3,5,7,8-pentamethyl-4-bora-3a,4a-diaza-*s*-indacene). Nuclei were counterstained with DAPI. Glycosaminoglycan deposition in chondrogenic differentiation was analyzed by Alcian Blue and PAS staining after 21 days of differentiation.

### RNA isolation and qRT-PCR analysis

RNA was isolated with the Nucleospin RNA Plus extraction kit (Macherey-Nagel), quantified with a NanoDrop ND-1000 spectophotometer (Thermo Scientific, Wathman, USA), and converted into cDNA with the high capacity cDNA reverse transcription kit (Life Technologies GmbH, Darmstadt, Germany). Semi-quantitative qRT-PCR was performed with TaqMan^TM^ gene expression master mix in a StepOnePlus machine (Applied Biosystems, Carlsbad, USA) using gene-specific TaqMan^TM^ assays (Table S2). Expression of glyceraldehyde 3-phosphate dehydrogenase (*GAPDH*) was used as reference.

### Gene expression profiles of iMSCs

Total RNA (200 ng per sample) was sequenced using a HiSeq 2500 system by Life & Brain GmbH (Bonn, Germany). Preprocessing of raw data was performed with SortMeRNA^68^ to exclude rRNA sequences and TrimGalore for adapter trimming. Reads were aligned with the STAR aligner^69^ and count tables were created with HTSeq^70^. Differential gene expression analysis was performed using the *R* package DESeq 2^71^. Differentially expressed genes were selected by adjusted t-test analysis (adjusted p-value for multiple testing <0.05). Gene Ontology analysis was performed with GoMiner^TM^. Raw data have been deposited at NCBI’s Gene Expression Omnibus (GEO, http://www.ncbi.nlm.nih.gov/geo/, accession number: GSE122545).

### DNA methylation profiles of iMSCs

Genomic DNA of iMSCs was isolated using the NucleoSpin Tissue kit (Macherey-Nagel). DNAm profiles were analyzed using the Infinium MethylationEPIC BeadChip Kit (Illumina) that covers more than 810,000 different CpG sites^72^. Bisulfite-conversion and subsequent hybridization of DNA (500 ng per sample) was performed at Life & Brain GmbH (Bonn, Germany). For further analysis, we excluded CpGs that did not vary across samples (standard deviation < 0.025) and utilized the unpaired limma-T-test to generate p-values that are adjusted for multiple testing with *R*. Raw data of DNAm profiles are accessible at GEO (accession number: GSE122469). Epi-Pluri-Score^45^ was calculated as described before.

### Statistics

All experiments were performed with three independent biological replicas, and results are presented as mean ± standard deviation (SD). Statistical significance was estimated by two-tailed paired Student’s t-test and t-test adjusted for multiple testing.

## Supplementary information

Supplementary Information

## Data Availability

Gene expression and DNA methylation profiles are available at Gene Expression Omnibus (GEO, http://www.ncbi.nlm.nih.gov/geo/) under the accession number GSE122546.
