# Supplementary material for: Differentiation of Induced Pluripotent Stem Cells towards Mesenchymal Stromal Cells is Hampered by Culture in 3D Hydrogels
Source: Sci Rep. 2019 Oct 30;9:15578. doi: 10.1038/s41598-019-51911-5 (PMC6821810; doi:10.1038/s41598-019-51911-5)
Supplement: Supplementary file 1 — Supplementary Information [file 41598_2019_51911_MOESM1_ESM.pdf]

Differentiation of Induced Pluripotent Stem Cells towards Mesenchymal Stromal Cells is Hampered by Culture in 3D Hydrogels

Roman Goetzke, Hans Keijdener, Julia Franzen, Alina Ostrowska, Selina Nüchtern, Petra Mela, Wolfgang Wagner

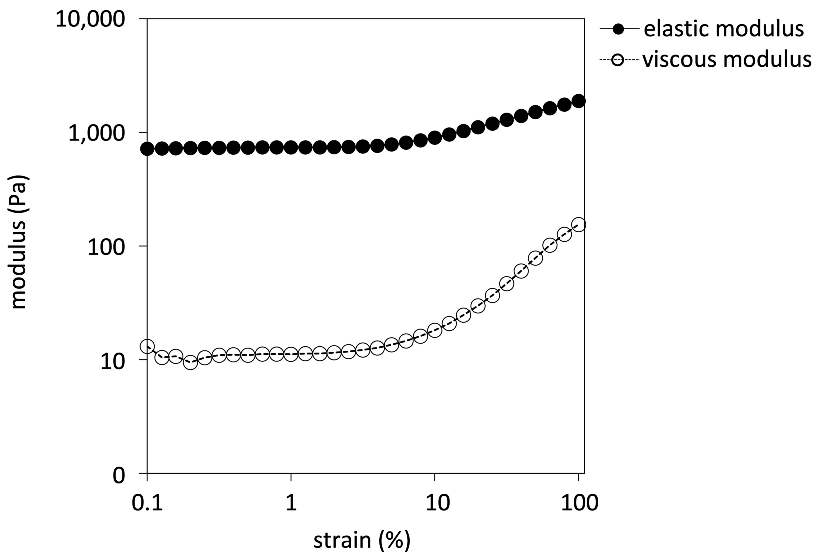

Figure S1. Rheological measurement of fibrin gel.
Rheological analysis of fibrin gel by strain sweep at 1 Hz and 0.1-100% strain. Fibrin gels demonstrated typical increase in elastic modulus (solid symbols) at higher strain. In contrast, viscous modulus (open symbols) was very low. Data represent measurements of four independent fibrin gel preparations at 37°C (mean values are depicted).

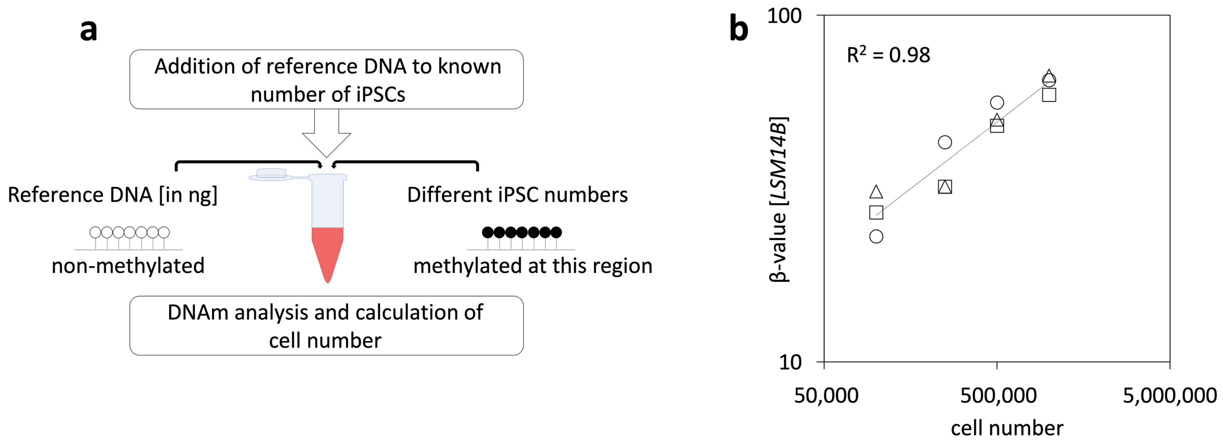

Figure S2. Cell quantification based on DNA methylation measurement.
(a) Schematic presentation of the method that is based on addition of a non-methylated reference DNA[1]. **(b)** Different cell numbers of iPSCs were mixed with the same amount of a reference plasmid comprising the non-methylated sequence for *LSM14B* (0.022 µg). DNAm levels at the relevant CpG site in *LSM14B* (cg06096175) were measured and plotted against the real cell number.

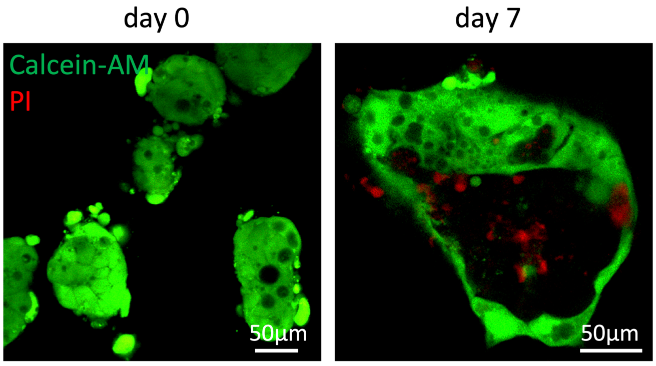

Figure S3. Apoptosis within iPSC-derived colonies inside hydrogel.
To analyze viability, iPSC-derived colonies were stained using Calcein-AM (living cells, green) and propidium iodide (PI; dead cells, red) at different time points during culture in fibrin gel. After one week of differentiation, iPSC colonies formed cavities filled with apoptotic cells.

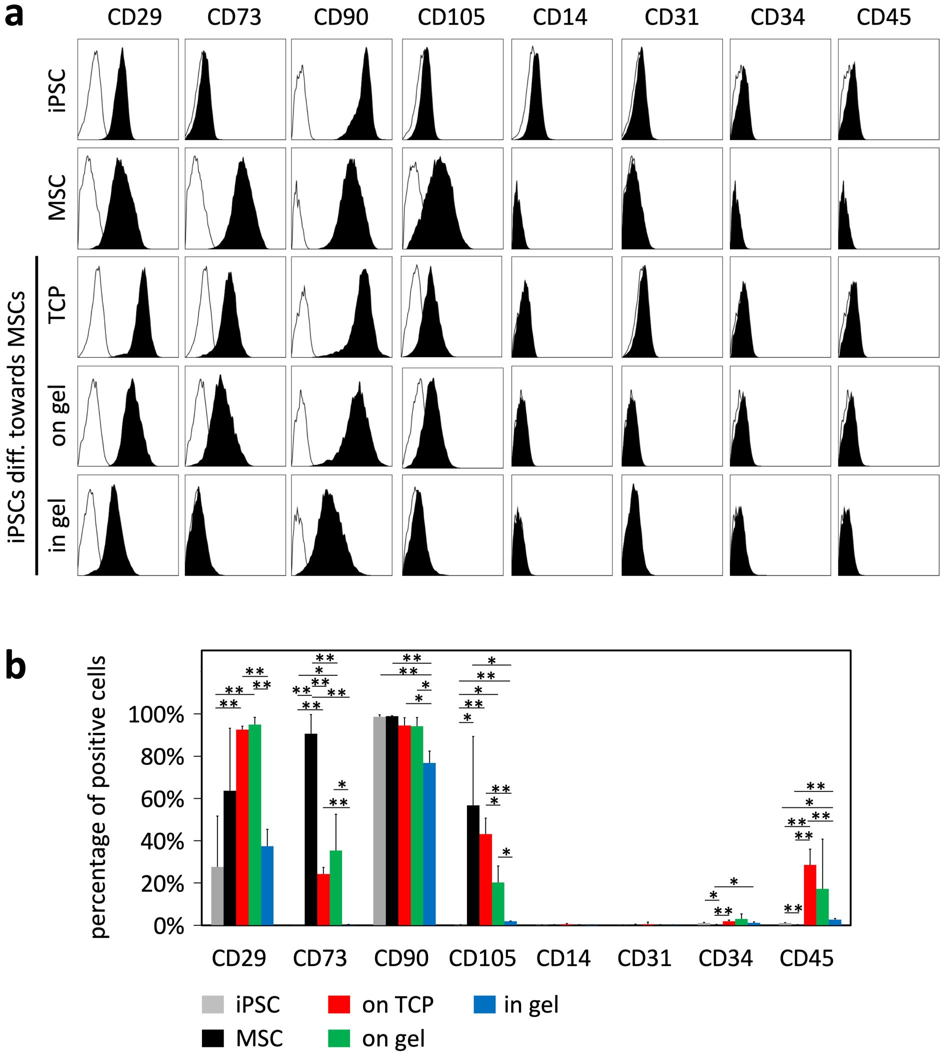

Figure S4. Immunophenotype of differentiated iPSC-derived cells in different culture conditions.
(a) Representative flowcytometric analysis after differentiation of iPSCs for 21 days. Representative measurements for MSCs and iPSCs are provided for comparison (autofluorescence indicated by black line). **(b)** Fraction of iPSCs differentiated for 21 days, which were positive for MSC-typical surface markers (n = 3, p-value adjusted for multiple testing: *p < 0.05, **p < 0.01).

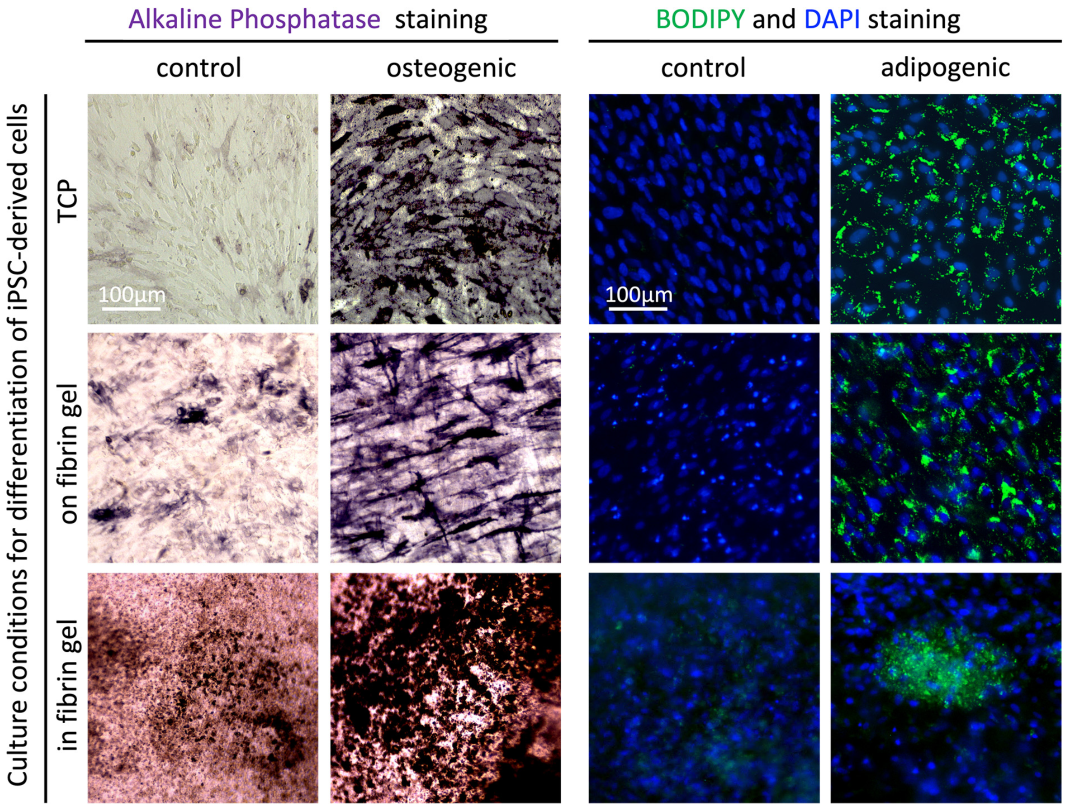

Figure S5. Osteogenic and adipogenic differentiation during culture in the three culture conditions. The iPSC-derived cells were differentiated into osteogenic and adipogenic lineage while the cells remained on TCP, on fibrin gel, or embedded within fibrin gel. After 14 days, osteogenic and adipogenic differentiation was assessed by alkaline phosphatase staining and BODIPY staining of fat droplets, respectively. In contrast to cell differentiation on TCP and on fibrin gel, inside fibrin gel there were hardly any areas with positive alkaline phosphatase staining or accumulation of intracellular fat droplets.

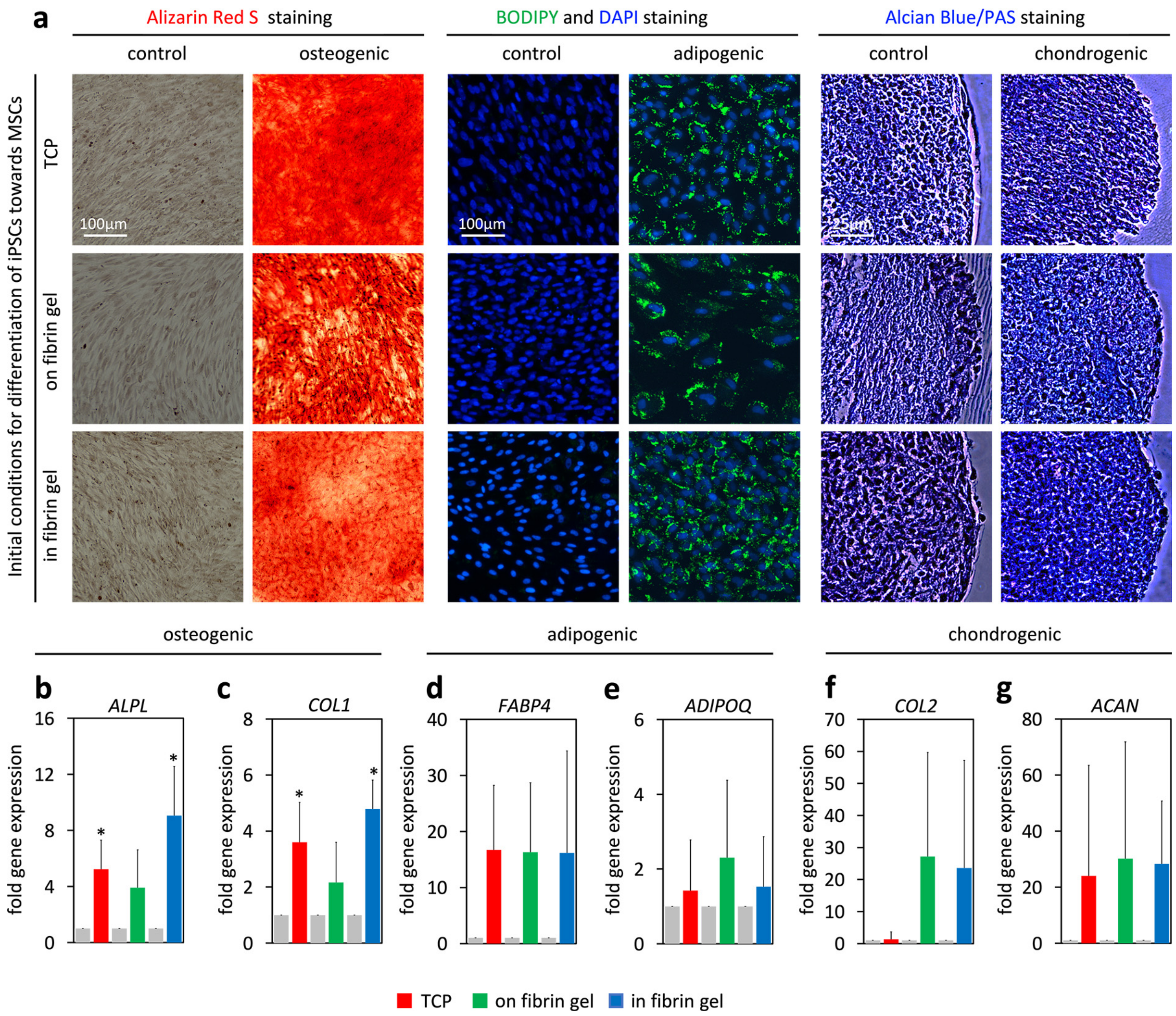

Figure S6. *In vitro* differentiation of iPSC-derived cells after harvesting from the three conditions. **(a)** After 21 days, the iPSC-derived cells were harvested from the different substrates and differentiated in parallel towards adipogenic, osteogenic, and chondrogenic lineages. After additional differentiation on TCP, all cell preparations revealed similar three-lineage differentiation potential as shown by staining of calcium precipitates by Alizarin Red S, fat droplets by BODIPY, and glycosaminoglycan deposition by Alcian Blue and PAS. **(b-g)** Furthermore, differentiation was analyzed by qRT-PCR of osteogenic, adipogenic, and chondrogenic marker genes (n = 3, *$p < 0.05$).

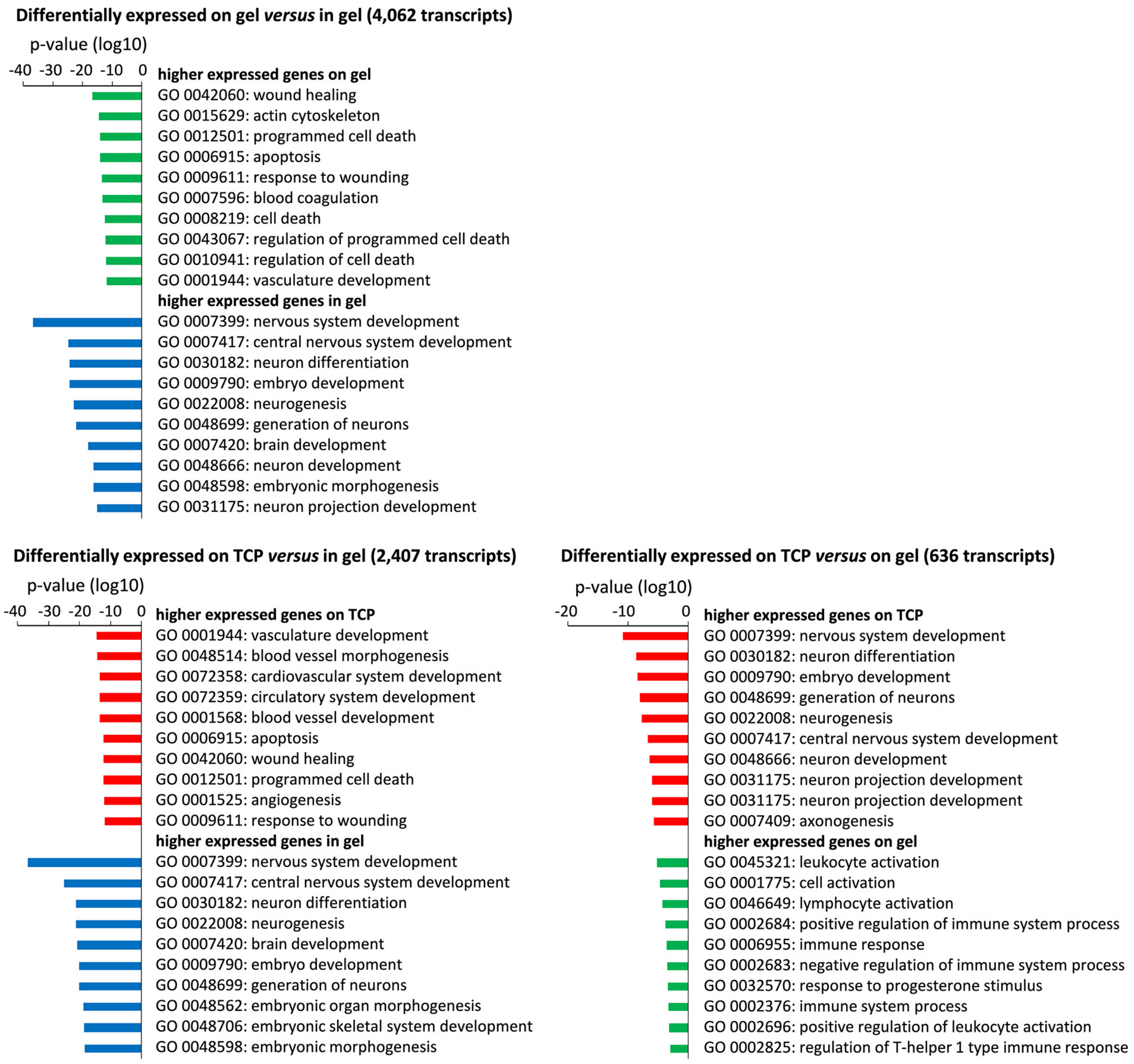

Figure S7. Gene Ontology classification of differentially expressed genes.
Pairwise comparison of cells that were differentiated for 21 days on tissue culture plastic, on fibrin gel, or within fibrin gel revealed significant gene expression differences (adjusted p-value < 0.05 and at least two-fold differential gene expression). These differences were classified by Gene Ontology analysis and enrichment in the most significant functional categories is depicted.

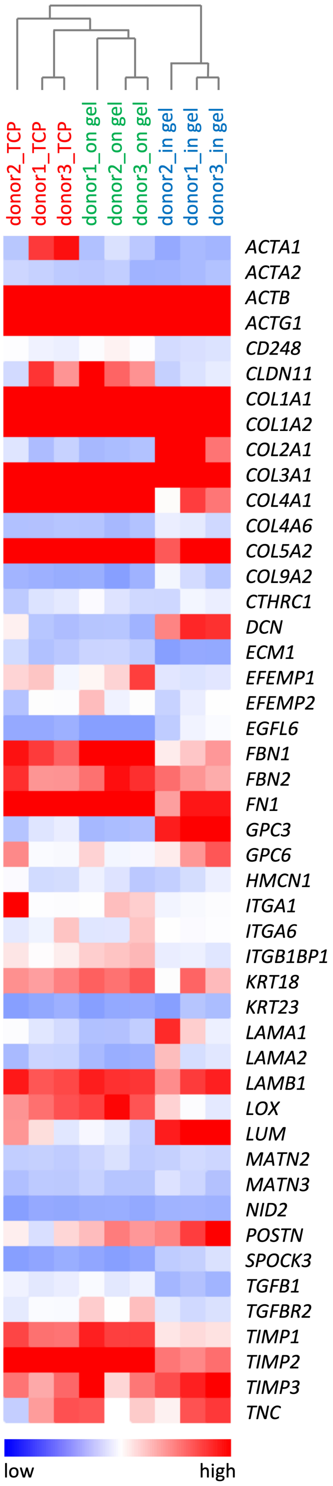

Figure S8. Genes for extracellular matrix are generally higher expressed on flat substrates.
The heatmap depicts the expression levels of genes associated with ECM. Overall, these genes are higher expressed on flat substrates. In addition, expression of actin genes is depicted (*ACTA1*, *ACTA2*, *ACTB*, *ACTG1*) and there was no clear difference between culture conditions.

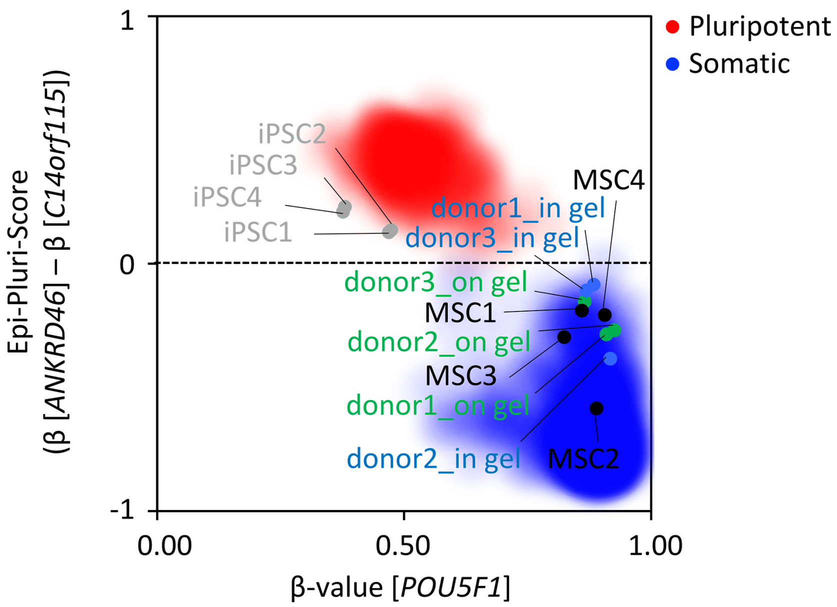

Figure S9. Classification of cell preparations with Epi-Pluri-Score.
DNA methylation was analyzed at three specific CpG sites. One of these CpGs was localized within the pluripotency-associated gene *POU5F1* (also known as *OCT4*). Furthermore, the difference in DNAm levels of CpGs in *ANKRD46* and *C14orf115* was determined and combined as Epi-Pluri-Score. The red and blue clouds refer to DNAm profiles of 264 pluripotent and 1,951 non-pluripotent cell preparations, respectively[2]. After 21 days of culture, either on or in fibrin gel, the iPSC-derived cell preparations were not classified as pluripotent anymore.

Table S1. Additional DNA methylation profiles that were used for comparison.

GEO ID	Cell type	Tissue of origin	# of samples	Accession codes of samples	Ref.
GSE113527	MSC	UC	4	GSM3108413, GSM3108414, GSM3108415, GSM3108416	3
GSE95531	iPSC		4	GSM2515899, GSM2515900, GSM2515901, GSM2515902	4

This table summarizes accession numbers for datasets at Gene Expression Omnibus (GEO; https://www.ncbi.nlm.nih.gov/geo/). DNA methylation profiles of these samples were all analyzed on the Infinium MethylationEPIC BeadChip (Illumina). MSC = mesenchymal stromal cell; iPSC = induced pluripotent stem cell; UC = umbilical cord; Ref = supplemental references as indicated below.

Table S2. Gene expression assays for qRT-PCR analysis.

Target gene	Full name	Assay ID
GAPDH	Glyceraldehyde 3-Phosphate-Dehydrogenase	Hs00164004_m1
COL1A1	Collagen Type 1 Alpha 1 Chain	Hs00164004_m1
COL2A1	Collagen Type 2 Alpha 1 Chain	Hs00264051_m1
ALPL	Alkaline Phosphatase	Hs00758162_m1
ACAN	Aggrecan	Hs00153936_m1
FABP4	Fatty Acid Binding Protein 4	Hs01086177_m1
ADIPOQ	Adiponectin	Hs00605917_m1

Supplemental References

1 Frobel, J. *et al.* Leukocyte counts based on DNA methylation at individual cytosines. *Clin Chem* **64**, 566-575 (2018).

2 Lenz, M. *et al.* Epigenetic biomarker to support classification into pluripotent and non-pluripotent cells. *Sci Rep* **5**, 8973 (2015).

3 De Witte, S. F. H. *et al.* Epigenetic changes in umbilical cord mesenchymal stromal cells upon stimulation and culture expansion. *Cytotherapy* **20**, 919-929 (2018).

4 Lee, J. H. *et al.* Lineage-Specific Differentiation Is Influenced by State of Human Pluripotency. *Cell Rep* **19**, 20-35 (2017).
